# The Correlation Between Meibomian Gland Dysfunction and Aniridia-Associated Keratopathy: A Prospective Analysis

**DOI:** 10.3390/jcm14030828

**Published:** 2025-01-27

**Authors:** Bogumił Wowra, Olga Łach-Wojnarowicz, Marzena Wysocka-Kosmulska, Dariusz Dobrowolski, Edward Wylęgała

**Affiliations:** 1 Chair and Clinical Department of Ophthalmology, Faculty of Medical Sciences in Zabrze, Medical University of Silesia in Katowice, 65 Panewnicka Street, 40-760 Katowice, Poland; olga.lachwojnarowicz@gmail.com (O.Ł.-W.); m.wysocka554@gmail.com (M.W.-K.); dardobmd@wp.pl (D.D.); wylegala@gmail.com (E.W.); 2 Department of Ophthalmology, District Railway Hospital, 65 Panewnicka Street, 40-760 Katowice, Poland

**Keywords:** aniridia, aniridia-associated keratopathy, meibomian glands, meibography

## Abstract

**Background/Objectives**: Aniridia is a rare congenital disorder characterized by structural and functional abnormalities in ocular development due to PAX6 haploinsufficiency, leading to complications such as aniridia-associated keratopathy (AAK). Meibomian gland dysfunction (MGD), a prevalent yet underexplored condition in aniridia, exacerbates tear film instability and chronic ocular surface inflammation, contributing to AAK progression. This study investigates the relationship between MGD severity and AAK in individuals with aniridia. **Methods**: This prospective randomized study included 113 participants (53 with aniridia and 60 controls). Comprehensive ophthalmic evaluations, including noninvasive meibography, were performed. The MGD severity was assessed using a standardized meiboscore scale, while the AAK severity was classified according to established clinical grading criteria. Statistical analyses, including Spearman’s correlation and chi-squared tests, were used to evaluate the relationships among MGD, AAK, and visual acuity. **Results**: MGD was significantly more prevalent and severe in the aniridia group compared to controls (*p* < 0.00001). A strong positive correlation was observed between MGD severity and AAK grade (r = 0.72, *p* < 0.00001), with both conditions associated with reduced best-corrected visual acuity (BCVA; r = −0.80 and −0.86, respectively, *p* < 0.0001). Age was positively correlated with MGD (r = 0.47, *p* = 0.0004) and AAK (r = 0.34, *p* = 0.0123), with gender-specific trends observed in females. **Conclusions**: MGD significantly contributes to AAK progression and visual impairment in aniridia. Meibography offers valuable insights into MGD severity, supporting early diagnosis and targeted interventions. Addressing MGD through tailored therapies could mitigate AAK progression and improve visual outcomes in this challenging condition.

## 1. Introduction

Aniridia is a rare, bilateral congenital condition characterized by incomplete or absent development of the iris and widespread ocular anomalies, with a prevalence estimated at approximately 1 in 50,000 to 100,000 live births [1]. The condition is predominantly caused by haploinsufficiency of the PAX6 gene, a highly conserved transcription factor essential for ocular morphogenesis and the maintenance of multiple eye structures [2]. This gene regulates the development of critical components such as the cornea, iris, retina, lens, and optic nerve. Consequently, mutations or deletions affecting PAX6 result in a spectrum of complex and multifaceted ocular abnormalities affecting both the anterior and posterior segments of the eye.

Beyond the hallmark feature of partial or complete absence of the iris, individuals with congenital aniridia face an elevated risk of progressive and sight-threatening complications. Corneal abnormalities are particularly common, with aniridia-associated keratopathy (AAK) developing due to limbal stem cell deficiency and chronic epithelial instability, ultimately leading to corneal scarring and opacification [3]. Additionally, cataracts, often present at birth or developing early in life, contribute significantly to visual impairment. Glaucoma, a challenging condition to manage in aniridia, can arise from anatomical anomalies in the angle and trabecular meshwork, leading to elevated intraocular pressure and optic nerve damage [4]. Optic nerve hypoplasia, another posterior segment manifestation, and nystagmus further compound the visual deficits associated with this condition. Collectively, these complications result in a progressive decline in visual acuity, necessitating lifelong, multidisciplinary management to preserve vision and improve the quality of life [5]. Comprehensive genetic counseling and early diagnostic interventions are critical for addressing the systemic and ocular implications associated with PAX6 mutations.

Aniridia-associated keratopathy (AAK) is one of the most complex and challenging complications linked to aniridia, a rare congenital disorder characterized by incomplete or absent iris development. AAK typically begins in childhood and progressively worsens over time, significantly impacting vision. The pathophysiology of AAK is multifaceted, involving a range of structural and functional abnormalities in the cornea. Key factors include corneal epithelial instability, damage to the basal cell layer, subepithelial fibrosis, chronic inflammation, and pathological neovascularization [5,6]. These processes lead to persistent corneal epithelial defects, scarring, and eventual corneal opacification, all of which contribute to a gradual and severe decline in visual acuity.

The progressive deterioration associated with AAK presents substantial clinical management challenges, as it often proves resistant to conventional therapeutic approaches. Current treatment strategies, including lubrication, anti-inflammatory medications, and surgical interventions, frequently offer limited long-term success, making comprehensive, personalized care essential. Emerging therapies such as regenerative medicine techniques, gene-based approaches, and stem cell transplantation are under investigation to better address the underlying mechanisms of AAK and improve patient outcomes [7,8].

Given its significant implications for patients’ quality of life, a multidisciplinary approach involving ophthalmologists, geneticists, and other specialists is crucial for optimizing care and support. Early diagnosis, continuous monitoring, and proactive management are key to mitigating the severe visual consequences of this condition. Ultimately, further research into the molecular mechanisms driving AAK will be vital in developing more effective, targeted treatment options.

An often-overlooked contributor to AAK is the role of ocular surface and tear film abnormalities, particularly meibomian gland dysfunction (MGD) [9]. The meibomian glands, situated within the tarsal plates of the eyelids, play a crucial role in maintaining ocular surface health. These specialized sebaceous glands produce and secrete lipids that form the outermost layer of the tear film. This lipid layer is essential for reducing tear film evaporation, thereby preserving the hydration of the ocular surface. Additionally, the lipids contribute to tear film stability, preventing breakdown and ensuring a smooth refractive surface, which is vital for clear vision [10]. MGD, which involves structural changes and functional impairments in these glands, is highly prevalent in aniridia patients and has been identified as a factor exacerbating ocular surface disorders and inflammation in AAK. Tear film instability resulting from MGD can exacerbate ocular surface dryness and evaporation, further aggravating the epithelial instability inherent in AAK and promoting a state of chronic inflammation. Consequently, understanding the relationship between MGD and AAK is crucial, as addressing MGD could offer a potential avenue for mitigating the progression of keratopathy in these patients [9,11].

One of the emerging tools for assessing MGD and ocular surface health is meibography, a noninvasive imaging technique that allows for detailed visualization and structural assessment of the meibomian glands. Through techniques such as infrared imaging, meibography can reveal important features of meibomian gland morphology, including dropout, atrophy, and irregular gland structure, which can be quantified to assess the severity of MGD [12]. In the context of aniridia, where alterations in gland function and tear film stability may accelerate the progression of AAK, meibography offers a valuable opportunity to characterize the underlying MGD and its potential contributions to the disease course. However, despite the clinical relevance of this imaging modality, there remains limited research specifically examining the role of meibography in patients with AAK. The objective of our study is to investigate and elucidate the potential correlations between MGD and AAK.

## 2. Materials and Methods

### 2.1. Study Population

This study adhered to the guidelines outlined in the Helsinki Declaration and was approved by the Bioethics Committee of the Silesian Medical University in Katowice (Approval No. KNW/0022/KB1/35/14, dated 16 May 2014). All participants provided informed consent before undergoing an examination and participating in the research. Random sampling was employed to select patients who had consented to participate between 2015 and 2024, resulting in a total of 53 subjects approved by the Bioethics Committee.

A prospective randomized study was conducted, including 52 participants diagnosed with aniridia. The cohort comprised 32 women and 28 men, with a mean age of 45.68 ± 14.74 years (range: 22–77 years). All participants underwent comprehensive ophthalmic evaluations, including meibography. A control group of 60 healthy individuals was also included. The exclusion criteria for the aniridia and the control group encompassed systemic diseases, dry eye disease (DED), the use of systemic or topical medications, prior ocular trauma or surgery, active ocular inflammation, a history of allergies, contact lens use, pregnancy, and breastfeeding. These strict criteria were applied to create highly uniform study and control groups, reducing potential confounding variables and strengthening the reliability of the research outcomes.

### 2.2. Meibography

**Figure 1:**
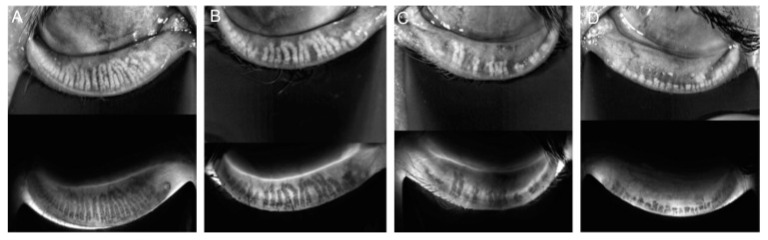
Meiboscore for grades 0–3. Notes: (**A**) Grade 0 indicates no loss of glands. (**B**) Grade 1 corresponds to gland loss of less than 33%. (**C**) Grade 2 represents gland loss ranging from 33% to 66%. (**D**) Grade 3 signifies gland loss exceeding 66%.

Meibography is a noninvasive imaging technique used to visualize the structure of the meibomian glands in vivo, providing valuable insights into glandular health and dysfunction. The system integrates a slit lamp equipped with an infrared (IR) transmitting filter and an IR charge-coupled device (CCD) video camera, allowing detailed imaging of the glands without the need for a transilluminating probe. This advanced setup enhances patient comfort and streamlines the assessment process. The evaluation of meibomian gland dropout, which refers to gland loss or structural alterations, was conducted by two trained assessors using a standardized meiboscore grading system. The meiboscore scale, ranging from 0 to 3, provides a consistent framework for quantifying glandular changes (Figure 1) [13,14].

The grading criteria are as follows:

- Grade 0 indicates no detectable gland loss.
- Grade 1 corresponds to gland loss involving less than one-third of the total glandular area.
- Grade 2 represents gland loss affecting between one-third and two-thirds of the gland area.
- Grade 3 denotes severe gland loss exceeding two-thirds of the total area [13].

For each participant, the meiboscore was determined by averaging the scores assigned by three independent graders for each eye. The mean scores from both eyes were then combined to generate a single, comprehensive meiboscore for each patient, reflecting the overall severity of glandular dysfunction [15,16]. This scoring system ensures objective and reproducible evaluation, facilitating the study of meibomian gland morphology and its relationship with ocular surface conditions such as meibomian gland dysfunction (MGD) and its impact on diseases like aniridia-associated keratopathy (AAK).

Noncontact meibography was performed on both upper and lower eyelids separately, using the Oculus Keratograph 5M (OCULUS Optikgeräte GmbH Münchholzhäuser Str. 29, 35582 Wetzlar, Germany) corneal topographic device with the Phoenix-Meibography Imaging software module. The patients were seated in front of the scanner with their foreheads resting against the headrest.

### 2.3. Aniridic Keratopathy

Aniridic keratopathy (AAK) is a progressive corneal disorder commonly associated with congenital aniridia. It is marked by a series of pathological changes in the corneal surface that impair vision and are challenging to manage. The key features of AAK include corneal surface damage, thinning or complete loss of the epithelial layer, chronic inflammation characterized by immune cell infiltration, abnormal vascularization, and progressive opacification of the cornea [6].

In the early stages of AAK, the limbal border, which plays a crucial role in maintaining corneal epithelial health by harboring limbal stem cells, may appear intact upon slit-lamp examination. However, as the disease advances, the integrity of the limbal border deteriorates, leading to a breakdown in barrier function. This is followed by the encroachment of conjunctival tissue near the limbus, signaling the onset of limbal stem cell deficiency (LSCD). Over time, the conjunctival tissue continues to grow over the peripheral and paracentral regions of the cornea, a process often accompanied by the development of vascular pannus—a fibrovascular tissue that further contributes to corneal clouding and vision loss.

In its most advanced stages, AAK results in complete corneal conjunctivalization, in which the entire corneal surface is replaced by conjunctival epithelium, causing extensive vascularization. The cornea transforms into a thickened, opaque structure, severely impairing visual function. These progressive changes underscore the need for early intervention and innovative therapeutic strategies, as conventional treatments provide limited efficacy in halting or reversing this complex degenerative process [17]. The classification of AAK is outlined in Table 1.

### 2.4. Statistical Methods

In the conducted studies, both quantitative and qualitative features were assessed. The analysis of the data presented and collected in this way has its specificity, consisting of the use of adequate statistical tools for the comparisons. The basic descriptive statistics were calculated in the form of arithmetic mean, median, standard deviation, skewness, and kurtosis. The Shapiro–Wilk test was used to verify the normality of the distribution. The nonparametric Spearman’s rank correlation coefficient was used to analyze the connections due to the lack of normality of the distribution and the rank nature of the variables. For the variables measured on rank and nominal scales, the counts and values of structure indices (percentages) were calculated and presented in graphs or tables. To verify whether there are connections between qualitative features, Pearson’s χ^2^ independence tests with NW correction or Yates’ χ^2^ for 2 × 2 tables were used.

The strength of the relationship was measured using Cramer’s V and Kendall’s tau b. The significance level of 0.05 was used for all analyses. All analyses were performed using the Statistica v.13.1 package and an Excel spreadsheet.

## 3. Results

In this study, 113 participants were evaluated, divided into a control group of 60 individuals (53.1%) and a study group of 53 individuals (46.9%). The groups were balanced by gender, with 32 females and 28 males in the control group and 28 females and 25 males in the study group, with no statistically significant gender-based differences between groups (*p* = 0.89).

The best-corrected visual acuity (BCVA) differed significantly between the control and study groups, with the mean BCVA being 0.95 in the control group and 0.046 in the study group (*p* < 0.0001). Nonparametric tests were used due to the non-normal distribution of the BCVA values.

The meibomian gland dysfunction (MGD) grades were significantly associated with group membership. In the control group, 83.3% exhibited no MGD (grade 0), compared to only 3.77% in the study group. Conversely, severe MGD (grade 3) was not observed in the control group but was present in 20.75% of the study group (*p* < 0.00001, V = 0.81). A gender-specific analysis revealed significant correlations in MGD grades for both males and females within the study group (*p* < 0.00001), but MGD grades were not significantly associated with gender itself (*p* > 0.05).

**Figure 2:**
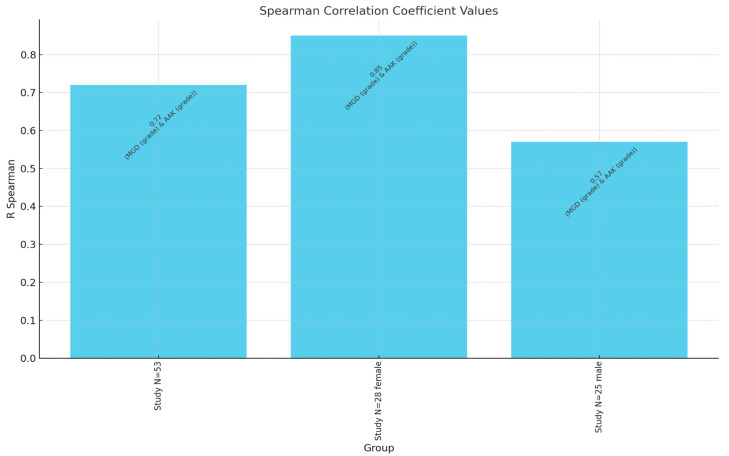
Spearman correlation coefficient values between MGD grade and AAK grade. N—number of patients.

The aniridia-associated keratopathy (AAK) grades showed no significant gender-based differences (*p* = 0.49). However, a strong positive correlation was identified between the MGD and AAK grades (r = 0.72, *p* < 0.00001), indicating that as the MGD severity increased, so did the severity of AAK. Age was also positively correlated with MGD severity (r = 0.47, *p* = 0.0004) and AAK severity (r = 0.34, *p* = 0.0123) in the overall study group. Among females, significant age correlations were observed with both MGD (r = 0.60, *p* = 0.0008) and AAK (r = 0.64, *p* = 0.0002). However, age did not significantly correlate with AAK in males (*p* = 0.71) (Figure 2).

**Figure 3:**
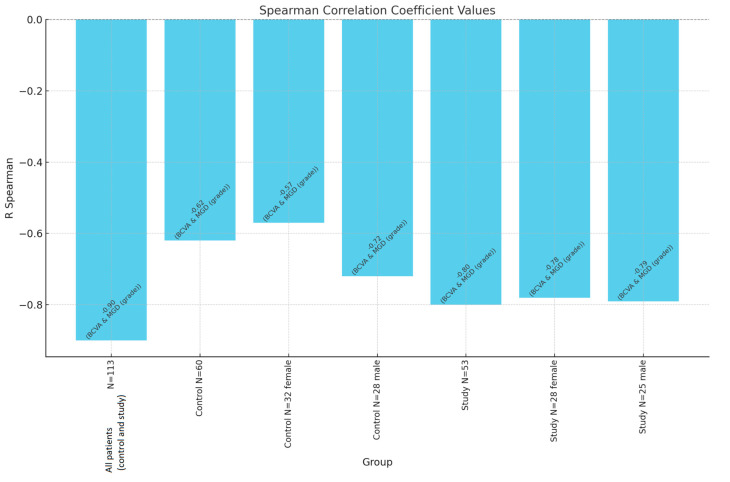
Spearman correlation coefficient values between BCVA and MGD grade. N—number of patients.

Finally, the BCVA demonstrated a strong negative correlation with both MGD (r = −0.80, *p* < 0.0001) and AAK (r = −0.86, *p* < 0.0001) within the study group, underscoring that greater disease severity was associated with poorer visual acuity. These findings highlight the interdependence of MGD, AAK, and age in the progression of visual impairment among individuals with aniridia (Figure 3).

## 4. Discussion

This study explores the complex interrelationship between meibomian gland dysfunction (MGD), aniridia-associated keratopathy (AAK), and visual impairment in patients with congenital aniridia, a rare genetic condition characterized by partial or complete absence of the iris. Our findings demonstrate a significant positive correlation between the severity of MGD and the progression of AAK, underscoring the critical importance of maintaining ocular surface health to mitigate corneal damage and preserve visual function in this patient population. The compromised tear film stability associated with MGD likely exacerbates corneal epithelial instability, contributing to the chronic inflammation and fibrosis observed in AAK.

Age emerged as a pivotal factor influencing the severity of both MGD and AAK. The older patients exhibited more advanced stages of these conditions, reflecting the progressive nature of corneal pathology over time. Furthermore, gender-specific trends were identified, suggesting potential hormonal influences or other physiological differences that warrant further investigation to optimize personalized treatment strategies.

These results provide valuable insights into the multifactorial nature of visual impairment in congenital aniridia. By highlighting the role of MGD in the progression of AAK, this study opens avenues for improved clinical management. Therapeutic strategies targeting MGD, such as warm compresses, lid hygiene, and meibomian gland expression, may offer benefits in slowing AAK progression. Additionally, the findings emphasize the need for routine ocular surface evaluation and age-appropriate interventions to preserve vision and enhance the quality of life of individuals living with aniridia.

This study stands out as the first to investigate the relationship between MGD and AAK in a Polish cohort of patients with aniridia. By focusing on this specific population, the research provides novel insights into the prevalence and progression of these conditions within a unique demographic, contributing valuable data to the global understanding of aniridia-associated ocular pathologies.

### 4.1. The Relationship Between MGD and AAK

Our study demonstrates that MGD is significantly more prevalent and severe in individuals with aniridia compared to healthy controls, with a strong correlation between MGD severity and AAK grade. This aligns with existing evidence suggesting that tear film instability and chronic ocular surface inflammation, exacerbated by MGD, play critical roles in the progression of keratopathy in aniridia [9,19,20]. The meibomian glands contribute essential lipids to the tear film, and dysfunction in these glands disrupts tear stability, increases evaporation, and fosters a pro-inflammatory environment on the ocular surface. Elevated inflammatory stress is evidenced by rising levels of inflammatory cytokines, that is TNF-α, IL-1, IL-6, and matrix metalloproteases. These inflammatory changes on the ocular surface trigger secondary effects, including modifications in the epithelial layer, such as squamous metaplasia. This, in turn, disrupts the population of goblet cells, which are crucial for maintaining the mucous component of the tear film, ultimately contributing to the damage and instability commonly observed in the tear film of affected patients [20,21,22].

In aniridia, these effects are compounded by inherent abnormalities in ocular development due to PAX6 haploinsufficiency. The absence or underdevelopment of the limbal stem cell niche and structural defects in the cornea exacerbate epithelial instability and susceptibility to inflammation [23,24]. MGD further accelerates this process by impairing the ocular surface’s ability to maintain homeostasis, thus contributing to the progressive damage observed in AAK.

Building on our findings of the significant interplay between MGD, AAK, and visual impairment in individuals with aniridia, the additional insights from studies on corneal nerve deficits and sensitivity further expand the understanding of ocular surface disease in this population [25,26]. Notably, the degradation of the tear film and ocular surface damage are not solely attributable to PAX6 mutations but are also influenced by broader mutation-independent factors such as deficits in corneal nerves and inflammatory dendritic cell activation [27]. These deficits, evident across all ages of aniridia patients, exacerbate tear film instability and ocular surface inflammation, particularly in older individuals or those with severe iris hypoplasia.

These findings suggest that interventions targeting nerve health, tear film restoration, and inflammation could bolster ocular surface homeostasis and slow the progression of AAK. The observed patterns of epithelial and corneal nerve degradation further highlight a critical therapeutic window early in life when the ocular surface remains relatively intact. Early intervention during this phase, potentially within the first decade, might prevent the onset of the vicious cycle of inflammation and structural degradation [3]. Moreover, considering the influence of iris hypoplasia on tear film stability and visual outcomes, tailored approaches accounting for the degree of hypoplasia could improve prognostic accuracy and treatment efficacy. These insights stress the importance of early, targeted strategies to preserve ocular health and mitigate progressive damage in aniridia-associated keratopathy [28].

### 4.2. Influence of Age and Gender

Age was positively correlated with the severity of both MGD and AAK, reflecting the progressive nature of these conditions. Interestingly, gender-specific trends were observed, with stronger age correlations in females. This difference may be attributed to hormonal influences on meibomian gland function, particularly the impact of androgens on lipid secretion. Previous studies have shown that androgens play a protective role in maintaining meibomian gland health, and age-related hormonal changes could disproportionately affect females [29,30]. On the other hand, Noland et al. [31] conducted a review focused on the literature regarding the prevalence of meibomian gland dysfunction (MGD) in relation to sex, with the goal of identifying whether women or men have a higher risk of developing MGD. A total of 24 relevant studies on the prevalence of MGD were reviewed, consisting of 10 population-based and 14 hospital-based studies. Among the population-based studies, five reported higher prevalence in men, three showed no significant difference, and one found higher prevalence in women. In the hospital-based studies, 10 reported no difference, two identified higher prevalence in men, and one found higher rates in women. Men may experience earlier and more severe meibomian gland changes, potentially linked to androgen decline, while women’s higher clinic attendance could bias hospital-based findings. Thus, further research is needed to explore these dynamics and develop targeted interventions.

### 4.3. Implications for Visual Acuity

The observed strong negative correlation between meibomian gland dysfunction (MGD), aniridia-associated keratopathy (AAK), and best-corrected visual acuity (BCVA) reflects the intricate relationship between ocular surface integrity and visual performance. MGD disrupts the lipid layer of the tear film, leading to instability, hyper-evaporation, and an inflammatory ocular environment, which exacerbates conditions like AAK. This cascade of damage not only worsens corneal clarity but also impairs neural and cellular interactions critical to visual acuity [25]. Recent hypotheses suggest that MGD-related dry eye may initiate or worsen visual degradation through multifaceted mechanisms. Inflammatory changes due to increased cytokine activity and alterations in meibum viscosity, common in MGD, degrade the tear film and ocular surface, contributing to epithelial and corneal nerve damage. These effects are compounded in conditions like AAK, where structural abnormalities from PAX6 mutations further destabilize the ocular surface [32,33].

### 4.4. The Role of Meibography in Clinical Practice

Meibography proved to be a valuable tool for quantifying MGD severity and identifying structural changes in the meibomian glands. Its noninvasive nature and ability to provide detailed imaging make it particularly suited for monitoring patients with aniridia, where chronic and progressive ocular surface disease is a major concern. Incorporating meibography into routine evaluations for aniridia patients could aid in identifying individuals at risk of severe MGD and allow for earlier intervention.

### 4.5. Future Directions

Given the significant interdependence of MGD and AAK, therapeutic strategies targeting MGD could play a pivotal role in managing aniridia-associated keratopathy. Treatments aimed at restoring meibomian gland function, such as thermal pulsation devices, intense pulsed light therapy, or lipid-based artificial tears, may help stabilize the tear film and reduce inflammation, thereby slowing the progression of AAK.

Additionally, further research is needed to elucidate the pathophysiological mechanisms linking MGD, tear film instability, and AAK. Longitudinal studies examining the efficacy of interventions targeting MGD in aniridia patients could provide valuable insights into optimizing care.

## 5. Conclusions

This study highlights the complex and multifactorial relationship between meibomian gland dysfunction (MGD) and aniridia-associated keratopathy (AAK) in individuals with congenital aniridia, emphasizing the critical role of ocular surface health in disease progression and visual impairment. Our findings demonstrate that MGD contributes significantly to tear film instability, chronic inflammation, and epithelial dysfunction, all of which exacerbate the severity of AAK. These insights reinforce the importance of a comprehensive, integrated approach to managing aniridia that prioritizes the early identification and treatment of ocular surface abnormalities. Addressing MGD alongside AAK is essential to slow the degenerative process and preserve corneal clarity and visual function over the long term.

The use of advanced diagnostic tools such as meibography has proven invaluable in objectively assessing meibomian gland structure and function. Incorporating this technology into routine clinical evaluations can facilitate early detection of glandular dropout and dysfunction, enabling timely and targeted interventions. The tailored treatment strategies that include thermal pulsation therapy, intense pulsed light, and lipid-based artificial tears show promise in restoring meibomian gland function, stabilizing the tear film, and reducing inflammation.

Furthermore, our findings point to the influence of age and gender on the severity of MGD and AAK, suggesting that hormonal and age-related factors should be considered when designing personalized treatment plans. Future longitudinal studies are needed to explore the long-term impact of MGD-targeted therapies on AAK progression and visual outcomes in congenital aniridia. By integrating these approaches, we can advance therapeutic outcomes, enhance quality of life, and provide a more hopeful prognosis for individuals affected by this challenging and complex ocular disorder.

## Figures and Tables

**Table 1:** Clinical classification of congenital aniridic keratopathy by Lopez-Garcia et al. [18].

Stage	Clinical Signs and Symptoms
Grade 0	Absence of signs and symptoms, but cytologic changes.
Grade 1	Pannus less than 1 mm. Fine epithelial staining with fluorescein-epiphora. Occasional epithelial defects.
Grade 2	Circumferential pannus with free visual axis. Photophobia, epiphora, lacrimal instability, and epithelial defects (two or more episodes in the last six months).
Grade 3	Stromal opacity with deposits and fibrosis affecting the corneal center. Chronic inflammation, marked neovascularization, and chronic epithelial defects.

## Data Availability

The data used to support the findings of this study are included in the article. The data will not be shared due to third-party rights and commercial confidentiality.

## References

[1] Calvão-Pires P., Santos-Silva R., Falcão-Reis F., Rocha-Sousa A. (2014). Congenital Aniridia: Clinic, Genetics, Therapeutics, and Prognosis. Int. Sch. Res. Not..

[2] Abdolkarimi D., Cunha D.L., Lahne M., Moosajee M. (2022). *PAX6* disease models for aniridia. Indian J. Ophthalmol..

[3] Ihnatko R., Eden U., Fagerholm P., Lagali N. (2016). Congenital Aniridia and the Ocular Surface. Ocul. Surf..

[4] Lee H., Khan R., O'Keefe M. (2008). Aniridia: Current pathology and management. Acta. Ophthalmol..

[5] Lagali N., Wowra B., Fries F.N., Latta L., Moslemani K., Utheim T.P., Wylegala E., Seitz B., Käsmann-Kellner B. (2020). PAX6 Mutational Status Determines Aniridia-Associated Keratopathy Phenotype. Ophthalmology.

[6] Latta L., Figueiredo F.C., Ashery-Padan R., Collinson J.M., Daniels J., Ferrari S., Szentmáry N., Solá S., Shalom-Feuerstein R., Lako M. (2021). Pathophysiology of aniridia-associated keratopathy: Developmental aspects and unanswered questions. Ocul. Surf..

[7] van Velthoven A.J.H., Utheim T.P., Notara M., Bremond-Gignac D., Figueiredo F.C., Skottman H., Aberdam D., Daniels J.T., Ferrari G., Grupcheva C. (2023). Future directions in managing aniridia-associated keratopathy. Surv. Ophthalmol..

[8] Vicente A., Sloniecka M., Liu J.X., Byström B., Pedrosa Domellöf F. (2022). Aniridia-related keratopathy relevant cell signaling pathways in human fetal corneas. Histochem. Cell Biol..

[9] Landsend E.C.S., Pedersen H.R., Utheim Ø.A., Xiao J., Adil M.Y., Tashbayev B., Lagali N., Dartt D.A., Baraas R.C., Utheim T.P. (2019). Meibomian gland dysfunction and keratopathy are associated with dry eye disease in aniridia. Br. J. Ophthalmol..

[10] Sabeti S., Kheirkhah A., Yin J., Dana R. (2020). Management of meibomian gland dysfunction: A review. Surv. Ophthalmol..

[11] Fries F.N., Moslemani K., Utheim T.P., Seitz B., Käsmann-Kellner B., Lagali N.S. (2023). Early ocular surface and tear film status in congenital aniridia indicates a supportive treatment window. Br. J. Ophthalmol..

[12] Swiderska K., Read M.L., Blackie C.A., Maldonado-Codina C., Morgan P.B. (2022). Latest developments in meibography: A review. Ocul. Surf..

[13] Kim C.K., Carter S., Kim C., Shooshani T., Mehta U., Marshall K., Smith R.G., Knezevic A., Rao K., Lee O.L. (2023). Risk Factors for Meibomian Gland Disease Assessed by Meibography. Clin. Ophthalmol..

[14] Fineide F., Arita R., Utheim T.P. (2021). The role of meibography in ocular surface diagnostics: A review. Ocul. Surf..

[15] Arita R., Itoh K., Inoue K., Amano S. (2008). Noncontact infrared meibography to document age-related changes of the meibomian glands in a normal population. Ophthalmology.

[16] Arita R. (2018). Meibography: A Japanese Perspective. Investig. Ophthalmol. Vis. Sci..

[17] Lagali N., Wowra B., Dobrowolski D., Utheim T.P., Fagerholm P., Wylegala E. (2018). Stage-related central corneal epithelial transformation in congenital aniridia-associated keratopathy. Ocul. Surf..

[18] López-García J.S., García-Lozano I., Rivas L., Martínez-Garchitorena J. (2006). Manejo terapéutico de la queratopatía asociada a aniridia congénita [Congenital aniridia keratopathy treatment]. Arch. Soc. Esp. Oftalmol..

[19] Jastaneiah S., Al-Rajhi A.A. (2005). Association of aniridia and dry eyes. Ophthalmology.

[20] Álvarez de Toledo Elizalde J., López García S., Benítez Del Castillo J.M., Durán de la Colina J., Gris Castejón O., Celis Sánchez J., Herreras Cantalapiedra J.M. (2021). Aniridia and the ocular surface: Medical and surgical problems and solutions. Arch. Soc. Esp. Oftalmol..

[21] Shiple D., Finklea B., Lauderdale J.D., Netland P.A. (2015). Keratopathy, cataract, and dry eye in a survey of aniridia subjects. Clin. Ophthalmol..

[22] Landsend E.C.S., Utheim Ø.A., Pedersen H.R., Aass H.C.D., Lagali N., Dartt D.A., Baraas R.C., Utheim T.P. (2018). The Level of Inflammatory Tear Cytokines is Elevated in Congenital Aniridia and Associated with Meibomian Gland Dysfunction. Invest. Ophthalmol. Vis. Sci..

[23] Polisetti N., Schlunck G., Reinhard T. (2023). PAX6 Expression Patterns in the Adult Human Limbal Stem Cell Niche. Cells.

[24] Chen S.Y., Cheng A.M.S., Zhang Y., Zhu Y.T., He H., Mahabole M., Tseng S.C.G. (2019). Pax 6 Controls Neural Crest Potential of Limbal Niche Cells to Support Self-Renewal of Limbal Epithelial Stem Cells. Sci. Rep..

[25] Lagali N., Edén U., Utheim T.P., Chen X., Riise R., Dellby A., Fagerholm P. (2013). In vivo morphology of the limbal palisades of Vogt correlates with progressive stem cell deficiency in aniridia-related keratopathy. Invest. Ophthalmol. Vis. Sci..

[26] Lagali N., Wowra B., Fries F.N., Latta L., Moslemani K., Utheim T.P., Wylegala E., Seitz B., Käsmann-Kellner B. (2020). Early phenotypic features of aniridia-associated keratopathy and association with PAX6 coding mutations. Ocul. Surf..

[27] Shetty R., Sethu S., Deshmukh R., Deshpande K., Ghosh A., Agrawal A., Shroff R. (2016). Corneal dendritic cell density is associated with Subbasal nerve plexus features, ocular surface disease index, and serum vitamin D in evaporative dry eye disease. Biomed. Res. Int..

[28] López-García J.S., Rivas L., García-Lozano I., Murube J. (2008). Autologous serum eyedrops in the treatment of aniridic keratopathy. Ophthalmology.

[29] Wang L.X., Deng Y.P. (2021). Androgen and meibomian gland dysfunction: From basic molecular biology to clinical applications. Int. J. Ophthalmol..

[30] Sullivan D.A., Sullivan B.D., Evans J.E., Schirra F., Yamagami H., Liu M., Richards S.M., Suzuki T., Schaumberg D.A., Sullivan R.M. (2002). Androgen deficiency, Meibomian gland dysfunction, and evaporative dry eye. Ann. N. Y. Acad. Sci..

[31] Nøland S.T., Magnø M.S., Utheim T.P., Chen X. (2024). Sex Differences in the Prevalence of Meibomian Gland Dysfunction: A Mini Review. Curr. Eye Res..

[32] Baudouin C., Messmer E.M., Aragona P., Greeling G., Akova Y.A., Benitez-del-Castillo J., Boboridis K.G., Merayo-Lloves J., Rolando M., Labetoulle M. (2016). Revisiting the vicious circle of dry eye disease: A focus on the pathophysiology of meibomian gland Dysfunction. Br. J. Ophthalmol..

[33] Teshigawara T., Akaishi M., Mizuki Y., Takeuchi M., Yabuki K., Hata S., Meguro A., Mizuki N. (2024). Dry Eye Treatment with Intense Pulsed Light for Improving Visual Outcomes After Cataract Surgery with Diffractive Trifocal Intraocular Lens Implantation. J. Clin. Med..

